# Insights for crystal mush storage utilizing mafic enclaves from the 2011–12 Cordón Caulle eruption

**DOI:** 10.1038/s41598-022-13305-y

**Published:** 2022-06-13

**Authors:** Heather Winslow, Philipp Ruprecht, Helge M. Gonnermann, Patrick R. Phelps, Carolina Muñoz-Saez, Francisco Delgado, Matthew Pritchard, Alvaro Amigo

**Affiliations:** 1 University of Nevada, Reno, Reno, USA; 2 Rice University, Houston, USA; 3 Departamento de Geología y Centro de Excelencia en Geotermia de los Andes (CEGA), FCFM, Universidad de Chile, Plaza Ercilla 803, Santiago, Chile; 4 Cornell University, Ithaca, USA; 5 Servicio Nacional de Geología y Minería (SERNAGEOMIN), Santiago, Chile

**Keywords:** Geochemistry, Mineralogy, Petrology, Volcanology

## Abstract

Two distinct types of rare crystal-rich mafic enclaves have been identified in the rhyolite lava flow from the 2011–12 Cordón Caulle eruption (Southern Andean Volcanic Zone, SVZ). The majority of mafic enclaves are coarsely crystalline with interlocking olivine-clinopyroxene-plagioclase textures and irregular shaped vesicles filling the crystal framework. These enclaves are interpreted as pieces of crystal-rich magma mush underlying a crystal-poor rhyolitic magma body that has fed recent silicic eruptions at Cordón Caulle. A second type of porphyritic enclaves, with restricted mineral chemistry and spherical vesicles, represents small-volume injections into the rhyolite magma. Both types of enclaves are basaltic end-members (up to 9.3 wt% MgO and 50–53 wt% SiO_2_) in comparison to enclaves erupted globally. The Cordón Caulle enclaves also have one of the largest compositional gaps on record between the basaltic enclaves and the rhyolite host at 17 wt% SiO_2_. Interstitial melt in the coarsely-crystalline enclaves is compositionally identical to their rhyolitic host, suggesting that the crystal-poor rhyolite magma was derived directly from the underlying basaltic magma mush through efficient melt extraction. We suggest the 2011–12 rhyolitic eruption was generated from a primitive basaltic crystal-rich mush that short-circuited the typical full range of magmatic differentiation in a single step.

## Introduction

**Figure 1:**
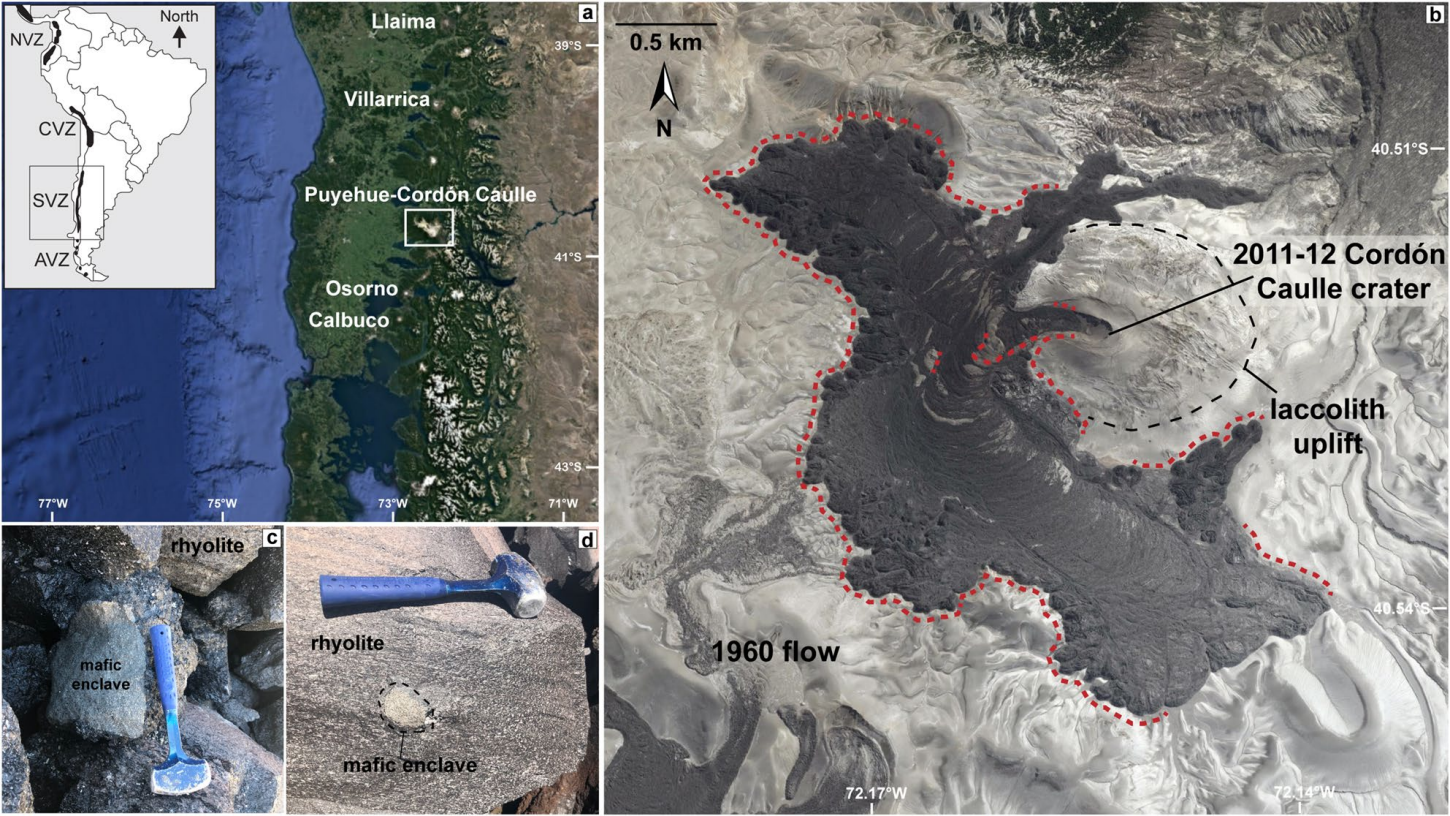
(**a**) Overview map of the SVZ of the Andes with inset of South America^15^. Overview map generated from Google Earth (Jan 2022; Image Landsat/Copernicus). White box identifies Puyehue-Cordón Caulle field area. (**b**) 2011–12 Cordón Caulle rhyolite lava flow. Image generated from Google Earth (Jan 2022; Image @ 2022 CNES/Airbus). Red dash: location of mafic enclave sampling for this study. Black dash: uplift area from laccolith^23^. (**c**,**d**) Field images of mafic enclaves hosted in rhyolite lava. Hammer for scale.

We have identified crystal-rich basaltic enclaves hosted in the rhyolite lava from the 2011–12 Cordón Caulle eruption located in the SVZ of Chile (Fig. 1). Here we report on the mafic enclave’s occurrence, their textural and geochemical characteristics, and we develop a conceptual model for their formation. Mafic enclaves are inclusions of chemically distinct, and typically crystal-rich, magma within a host magma that tends to be compositionally more evolved than the inclusion^1^. Mafic enclaves can form by a spectrum of processes that result in a more or less direct genetic and spatial link between the enclave-forming mafic magma and the more evolved host magma. Mafic enclaves are most commonly associated with magma recharge into an already established or unrelated magmatic system^1–4^. In contrast, based on textural and geochemical characteristics, the 2011–12 Cordón Caulle enclaves likely represent pieces of a crystal-rich magma mush that were incorporated into the crystal-poor rhyolite magma erupted at Cordón Caulle. In the case of a crystal mush origin, the mafic enclaves may also provide direct insight toward silicic magma production.

Crystal mushes are defined as large, crystal-dominated (~ 45–65%) storage reservoirs that contain evolved interstitial melt^5–7^. Previous studies have highlighted that crystal-rich magma mush storage reservoirs can produce voluminous and explosive eruptions of silicic magma, sometimes up to > 5000 km^3^ such as that of Fish Canyon Tuff^5–8^. They are associated with large caldera-forming eruptions, such as the Bishop Tuff at Long Valley Caldera, as well as volumetrically smaller systems such as Mount St. Helens and Mt. Mazama^3,6,9^. A crystal mush is more mafic in bulk composition than the evolved interstitial melt, and if the interstitial melt is extracted, it can form an overlying crystal-poor silicic melt lens cap^5,6,10,11^. Fractional crystallization is considered to be the dominant process to generate such crystal-poor silicic magma in crystal mushes^12,13^. This is likely associated with the high crystallinity of crystal mushes that allows for increased residual melt evolution^6,12,13^. While most crystal-rich magma mushes are intermediate in composition, thus making a step-wise change from intermediate to felsic magmas when producing the melt lens cap^5–7^, Cordón Caulle may contain a basaltic mush based on the basaltic mafic enclaves. This means Cordón Caulle may experience highly efficient fractional crystallization to produce rhyolite directly from basalt. Additionally, if the mafic enclaves are representative of a crystal mush, they represent evidence of an active crystal mush and will provide new opportunities to study mush dynamics at an active volcanic system. Whether a crystal mush architecture can efficiently generate large volumes of eruptible silicic magma directly from a basaltic crystal-rich mush has not yet been demonstrated in volcanic arcs. This study highlights how mafic enclaves can reveal processes in active magmatic systems other than mafic recharge. Here we argue that the Cordón Caulle mafic enclaves are evidence for an underlying crystal mush as opposed to representing magma recharge, and that the enclaves may constrain the efficiency and limits to fractional crystallization and rhyolite formation as well as provide global insight for crystal mush dynamics in volcanic arcs.

## Background

Cordón Caulle is a fissure system amidst a NW-elongated graben that is part of the larger Puyehue-Cordón Caulle volcanic complex (PCC; 40.31° S, 72.10° W), which is located in the Chilean SVZ^14,15^. PCC is a laterally extensive volcanic complex comprised of the stratocone Puyehue, the 15 km-long Cordón Caulle fissure system, and the Cordillera Nevada Caldera, a collapsed shield volcano^16^. Cordón Caulle has been the site of three eruptions during the last 100 years (1921–22, 1960, 2011–12)^14,15,17–19^. Multiple eruptive vents for the 1921–22 and 1960 eruptions are distributed along faults, while the 2011–12 eruption was restricted to a singular eruptive vent. All three eruptions produced dacitic to rhyolitic lava flows of approximately 1 km^3^ and distributed ash regionally^15,17–19^. Published geothermobarometry data suggests that shallow magma storage is associated with all three of the historic eruptions (50–100 MPa and 870–920 °C^17^; 5–7 km, 100–140 MPa, and 895 °C^18^; 3–7 km^20^.

Satellite- and ground-based monitoring have provided exceptional pre-, syn-, and post-eruptive deformation across the entire Cordón Caulle graben for the 2011–12 eruption^21–23^. Inflation of approximately 0.5 m preceded the eruption for several years, subsidence of approximately 4.5 m was recorded during the eruption, and rapid re-inflation of approximately 1 m followed the eruption, which represents one of the largest uplift rates for silicic systems with ~ 0.45 cm/year immediately after the eruption^21,22,24^. Additional localized inflation beneath the 2011–12 vent has been interpreted as rapid laccolith emplacement shortly after the onset of the eruption (Fig. 1)^23^. The laccolith is located ~ 20–250 m below the surface and the overlying volcanic deposits have extensive cracks and fumarolic activity in the vicinity of the vent area^23^. In recent years, inflation has been episodic and because the ground deformation extends over the entire Cordón Caulle graben, it suggests a spatially connected and laterally extensive magmatic body that accommodates magma redistribution, either in response to recharge or post-eruptive poro-elastic effects within a mush stored in the shallow crust^21,22,24^. A crystal-mush model has been proposed in previous studies due to the spatially distributed eruptive vents and deformation signals, but here we present direct evidence for a crystal-rich magma mush through petrologic and geochemical analysis of mafic enclaves^15,17–19^.

## Results

### Enclave petrography

**Figure 2:**
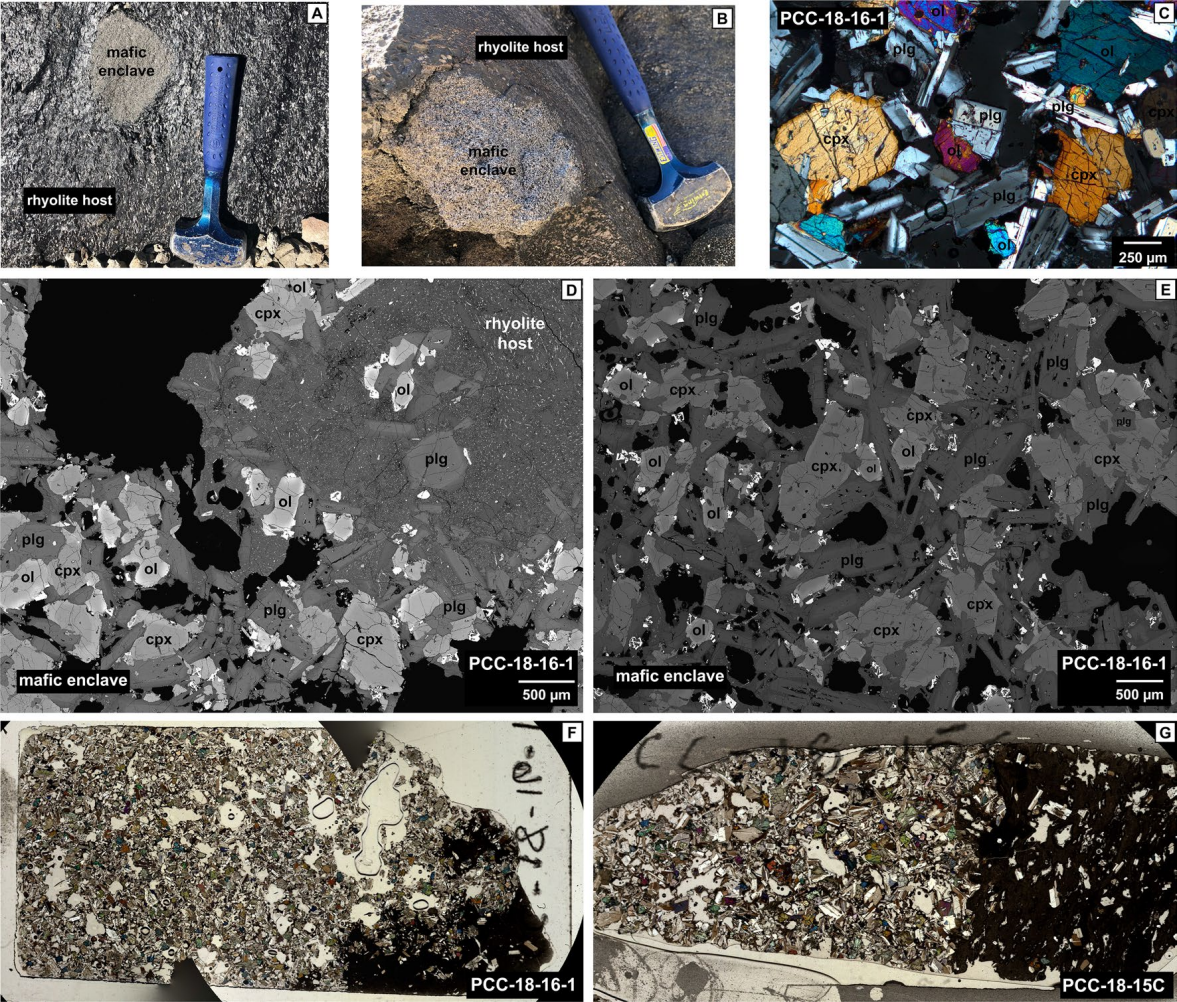
Textural images of the coarsely-crystalline enclave population. (**a**,**b**) Field photos of mafic enclaves in rhyolite host with hammer for scale. (**c**) Micrograph highlighting enclave mineralogy, crystallinity, and interlocking grain texture. (**d**) Backscattered electron (BSE) map of mafic enclave-rhyolite host boundary. (**e**) BSE map of enclave core. (**f**,**g**) Thin section scans of coarsely-crystalline enclaves highlighting crystallinity, mineralogy, and enclave-host boundary.

**Figure 3:**
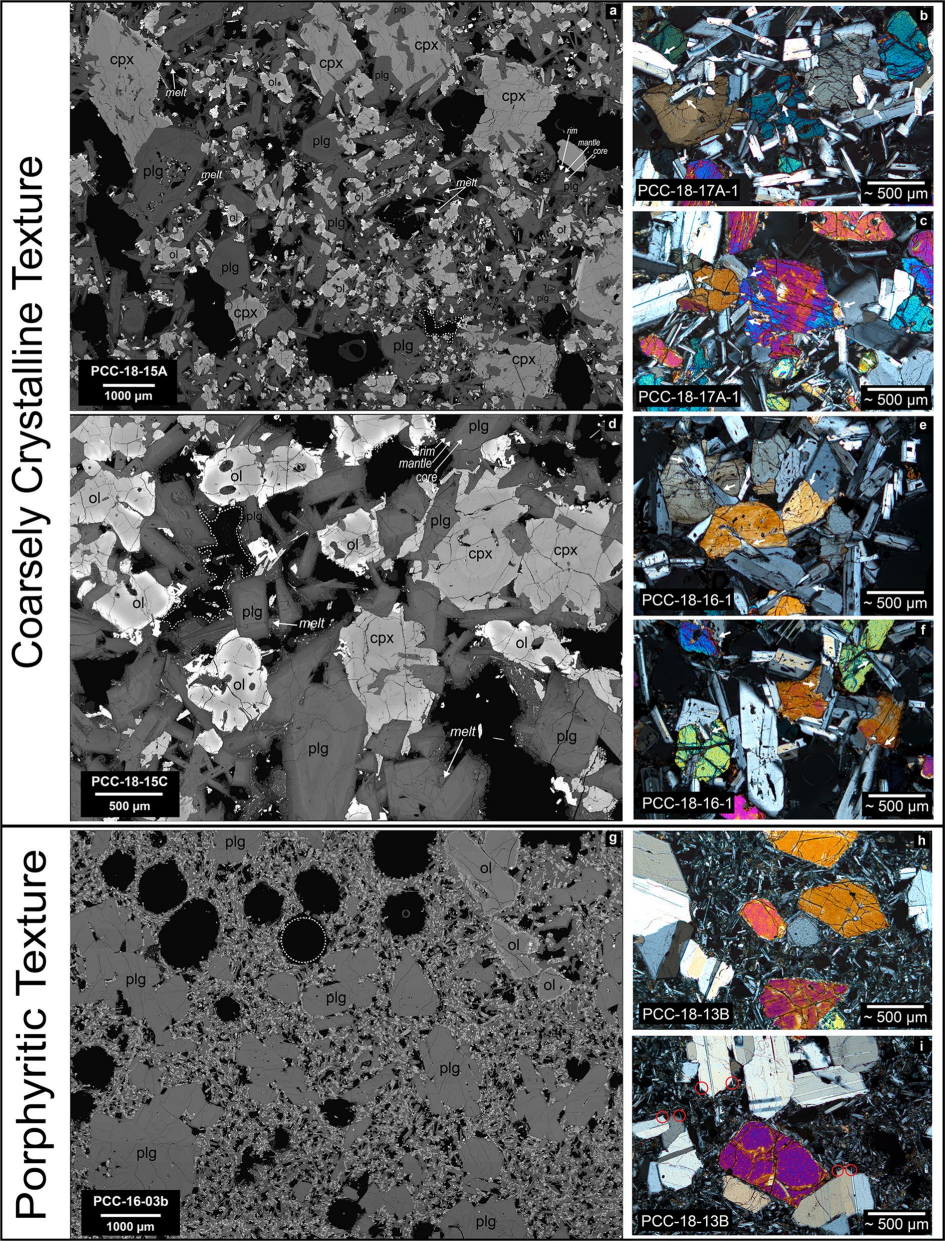
(**a**–**i**) Textural comparison of the coarsely-crystalline and porphyritic enclave populations using BSE maps and micrographs. Images highlight variations in crystallinity and vesicle shape differences with spherical vesicles in porphyritic enclaves and angular void-filling vesicles in the coarsely-crystalline population. Dotted white lines: highlight vesicle shapes in both enclave populations (**a**,**d**,**g**). (**b**,**c**,**e**,**f**) Micrographs display interlocking grain textures that are highlighted by white arrows. (**i**) Micrograph of porphyritic enclave with plagioclase clusters. Red circles highlight swallowtail disequilibrium textures of plagioclase.

Two types of mafic enclaves are present in the 2011–12 rhyolite flow. The majority of enclaves are relatively coarsely-crystalline dominated by phenocrysts (~ 55–70 vol%) (Fig. 2), while a subordinate group of enclaves (2 of 33 samples) are distinctly porphyritic with < 35 vol% phenocrysts (Fig. 3; Table 1; ESM 2). Both types are vesiculated, yet the coarsely-crystalline enclaves contain a slightly greater abundance of vesicles (> 15 vol%) compared to the porphyritic enclaves (< 15 vol%) (Fig. 3, Table 1). While technically the dominant enclave population is fine- to medium-grained, we will refer to them as “coarsely-crystalline” as a relative comparison to the porphyritic population. Mafic enclaves range in size from ~ 5 to 20 cm in diameter, but occasionally reach up to ~ 40 cm (Fig. 2a,b). Mafic enclaves are sub-rounded to sub-angular and are commonly found in devitrified rhyolite. We observe occasional pressure shadows surrounding the enclaves that suggests they were mostly solid during the lava emplacement^25–27^.

**Table 1:** Crystallinity of mineral phases, microlites, melt, and vesicles for both enclave populations. All values represent percentages (%).

Sample	Coarsely-crystalline enclaves					Porphyritic enclaves	
	PCC-18-15A	PCC-18-15C	PCC-18-16-1	PCC-18-17A-1	PCC-18-17D-1	PCC-18-13B	PCC-16-03B
Phenocryst crystallinity	66	67	62	70	55	35	32
Plagioclase	37	40	40	45	45	32	28
Olivine	13	16	6	9	6	2	4
Clinopyroxene	16	11	16	16	4	1	0
Microlites	10	11	10	10	27	54	54
Melt	6	4	2	5	1	–	–
Vesicles	18	18	26	15	17	11	14
Total	100	100	100	100	100	100	100

#### Coarsely-crystalline enclaves

The coarsely-crystalline enclaves are characterized by interlocking textures with average phenocryst sizes ranging from 300 to 600 µm but frequently reach > 1000 µm (Figs. 2, 3). Phenocryst phases are listed in order of abundance: plagioclase (~ 40–45 vol%), clinopyroxene and olivine (~ 5–15 vol% with varying proportions), and Fe–Ti oxides (~ < 1 vol%) with accessory apatite (Table 1). Plagioclase microlites dominate the groundmass (~ 10 vol%), and small amounts of melt pockets, now solidified glass, (~ 1–5 vol%) are present at the edges of phenocryst phases (Fig. 3a,d; Table 1; ESM 3, 4). The pervasive intergrowth and poikilitic textures between plagioclase, olivine, and clinopyroxene suggest coeval growth (Figs. 2, 3). Microlitic plagioclase also experiences impingement and intergrowth into phenocryst phases. The varying degrees of interlocking phases between microlites, microphenocrysts, and large phenocrysts (Figs. 2, 3; ESM 3, 4) may indicate bursts of nucleation at varying nucleation and growth rates^28^. Phenocrysts and microlites do not present lineation or foliation fabrics and therefore do not exhibit settling or compaction features. Low aspect ratios of phenocrysts (i.e. equant crystal shapes) is suggestive of slow cooling as opposed to rapid cooling typical for small dikes that may form more elongate crystal shapes due to greater undercooling^28,29^. While phenocrysts of plagioclase and clinopyroxene are subhedral and display planar surfaces on edges exposed to the groundmass, they are dominantly intergrown with other well faceted phenocrysts at random orientations (Fig. 3). Melt pockets surrounding phenocrysts are irregular in shape and do not display parallel sides (Fig. 3; ESM 3, 4). Vesicles display irregular void-filling shapes amidst the crystal intergrowth (Fig. 3a,d). Generally, quench textures and chilled margins are absent in the coarsely-crystalline enclaves and crystal size and glassiness of the matrix do not vary systematically from interior to exterior. However, some groundmass heterogeneities near the enclave-host interface exist with distinct heterogenous strands of groundmass and small changes in microlite size and vesiculation in the rhyolite (ESM 5, e.g., PCC-18-15C). Enclave margins display a moving boundary with crystal clusters and glomerocrysts seemingly breaking off from the enclave into the rhyolite or being individually plucked into the rhyolite.

Plagioclase phenocrysts and microlites are normally zoned, the former sometimes exhibiting varying degrees of sieve textures within their cores (Figs. 2, 3; ESM 3). Plagioclase phenocrysts display stepwise zonation in anorthite (An) content. The calcic cores are homogeneous (~ An_80–90_) with only small normal and reverse internal zonation (ΔAn < 5), followed by a distinct step in An content (An_55–65_, ~ 30 µm width). The outermost plagioclase has even lower An contents (< 10 µm width) (ESM 3). We refer to these distinct zones as the core, mantle, and rim (Fig. 3a,d; ESM 3). Microlites exhibit both elongate and equant habit, and their zonation displays core-rim normal zonation without a significant mantle. Olivine phenocrysts have flat cores^30^ with weak normal zonation limited to about the outermost 100 µm. Olivine in contact with other minerals can be unzoned. Anhedral olivine can be found as chadacrysts amidst plagioclase oikocrysts (ESM 3, 4). Clinopyroxene phenocrysts exhibit simple zonation with distinct compositional steps near their rims. In a few enclaves, clinopyroxene phenocrysts are the largest phenocryst phase with bimodal size distributions. As an example, PCC-18-15A contains populations of larger and smaller clinopyroxene with sizes of > 1000 µm and < 500 µm, respectively (Fig. 3a), the latter being more frequent. Cores of larger clinopyroxene phenocrysts commonly contain plagioclase inclusions (ESM 3a, d). Olivine and clinopyroxene display frequent orthopyroxene reaction rims when in contact with the melt and not bounded by other phenocrysts (notated in ESM 4c,d,g,j,k). Peritectic orthopyroxene reaction rims are a late-stage process that are likely the result of interacting with the more evolved rhyolitic liquid^31^. Fe–Ti oxides exhibit frequent skeletal and dendritic growth.

Glomerocrysts occur throughout the rhyolite and near the enclave-rhyolite interface. Two populations of glomerocrysts have been identified. The first population resembles the same mineralogy, composition, and zonation patterns of the coarsely-crystalline enclaves. They are present at the enclave-host boundary. In this population, plagioclase has core-mantle-rim zones similar to plagioclase in coarsely-crystalline enclaves. These glomerocrysts seemingly represent broken off enclave fragments. The second glomerocryst population is not associated with the enclaves and only occurs in the rhyolite. It consists of plagioclase, clinopyroxene, olivine, and frequent Fe–Ti oxides as inclusions in clinopyroxene and olivine. These glomerocrysts have distinct composition and zonation patterns with plagioclase phenocrysts being uniformly evolved and minimally zoned in the interior. Rims have similar An contents to plagioclase rims in the enclaves (ESM 5). Fe–Ti oxides range from tens of microns up to ~ 150 µm and exhibit blocky habit (ESM 5). Additionally, exsolution lamellae can be identified in clinopyroxene glomerocrysts. The distinct textures of the second population of glomerocrysts suggest direct entrainment into the rhyolite independent of enclave formation.

#### Porphyritic enclaves

The second less common enclave population displays porphyritic textures with a very distinct groundmass (Fig. 3g–i). Phenocrystic plagioclase (~ 30%) dominates the mineral assemblage. Olivine (~ 2%), clinopyroxene (< 1%), and oxides are subordinate (Table 1). The porphyritic enclaves have characteristic large spherical vesicles (Fig. 3g) as opposed to the coarsely-crystalline enclaves, whose vesicles are void filling and their irregular shapes are controlled by the crystal network. Plagioclase phenocrysts are dominantly large (> 1000 µm) and display both elongate and equant crystal shapes with infrequent sieve textures. A subordinate plagioclase population has smaller crystal sizes (~ 250–500 µm). Regardless of size, plagioclase is commonly arranged in glomerocrysts. Plagioclase grain boundaries that are in contact with groundmass exhibit swallowtail textures (Fig. 3i; ESM 2e,f). Plagioclase core compositions are uniform with weak normal zonation at the outermost rim that is significantly less developed compared to coarsely-crystalline enclaves. Both size populations of plagioclase display similar compositional and zonation patterns. Olivine phenocrysts (~ 1000 µm) display both anhedral and skeletal crystal shapes (ESM 2c,d) with flat core compositions and orthopyroxene overgrowth^30^. Groundmass (~ 55%) consists of plagioclase microlites (70–100 µm) and interstitial mafic phases.

### Geochemistry

#### Whole-rock data

**Figure 4:**
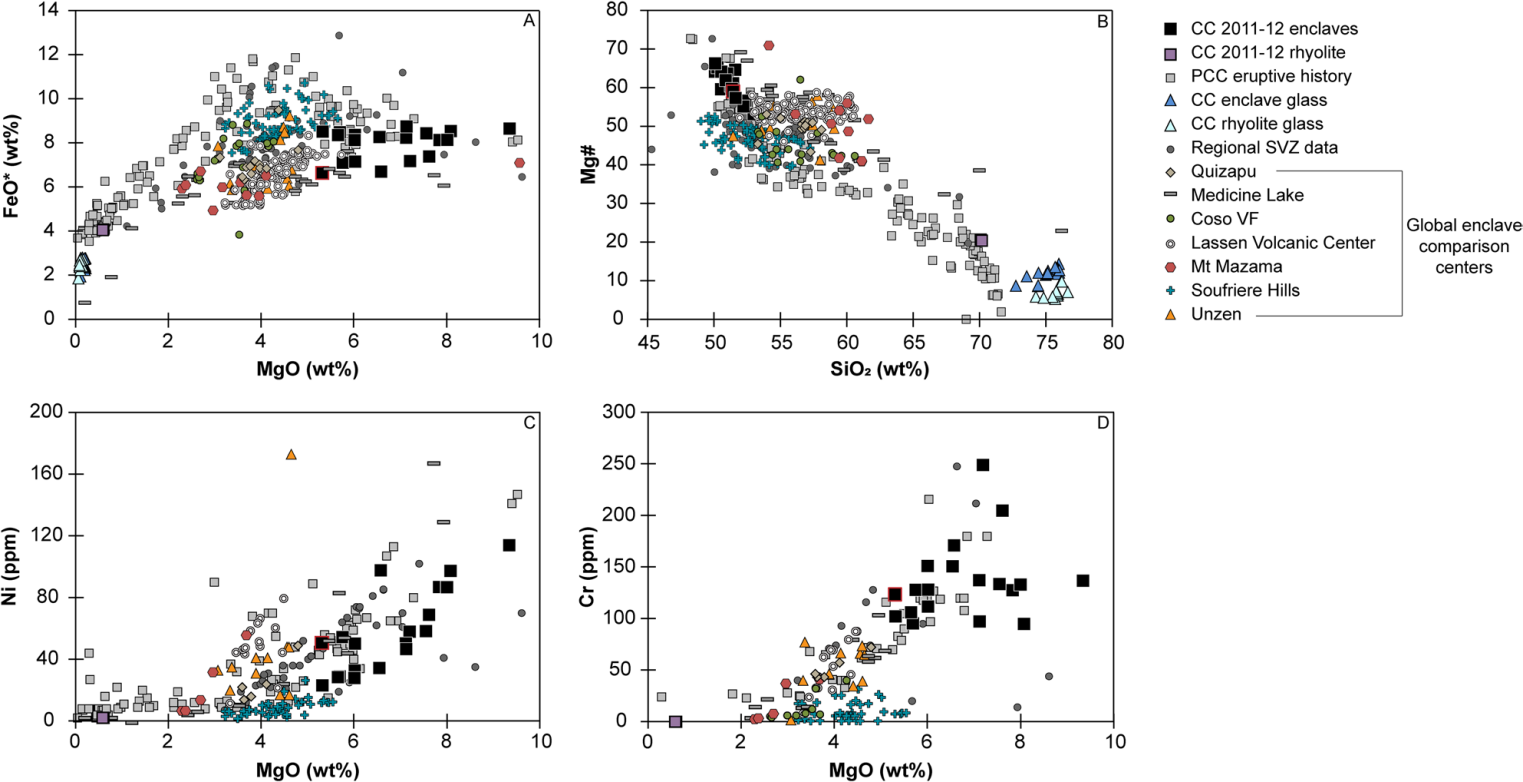
Global geochemical compilation of mafic enclaves (XRF data). 2011–12 CC enclaves are at primitive extent. CC: Cordón Caulle. Black square: 2011–12 CC coarsely-crystalline mafic enclaves. Black square with red outline: indicates porphyritic enclave population among CC enclaves. Purple square: 2011–12 CC rhyolite. Grey square: PCC eruptive history (EarthChem). Triangles: 2011–12 CC enclave and rhyolite glass (EMP data). Dk Grey circle: Regional SVZ data (EarthChem). Global enclave comparison centers: Quizapu, Medicine Lake, Coso VF, Lassen Volcanic Center, Mt Mazama, Soufriere Hills, Unzen. References for global centers in ESM 1.

**Figure 5:**
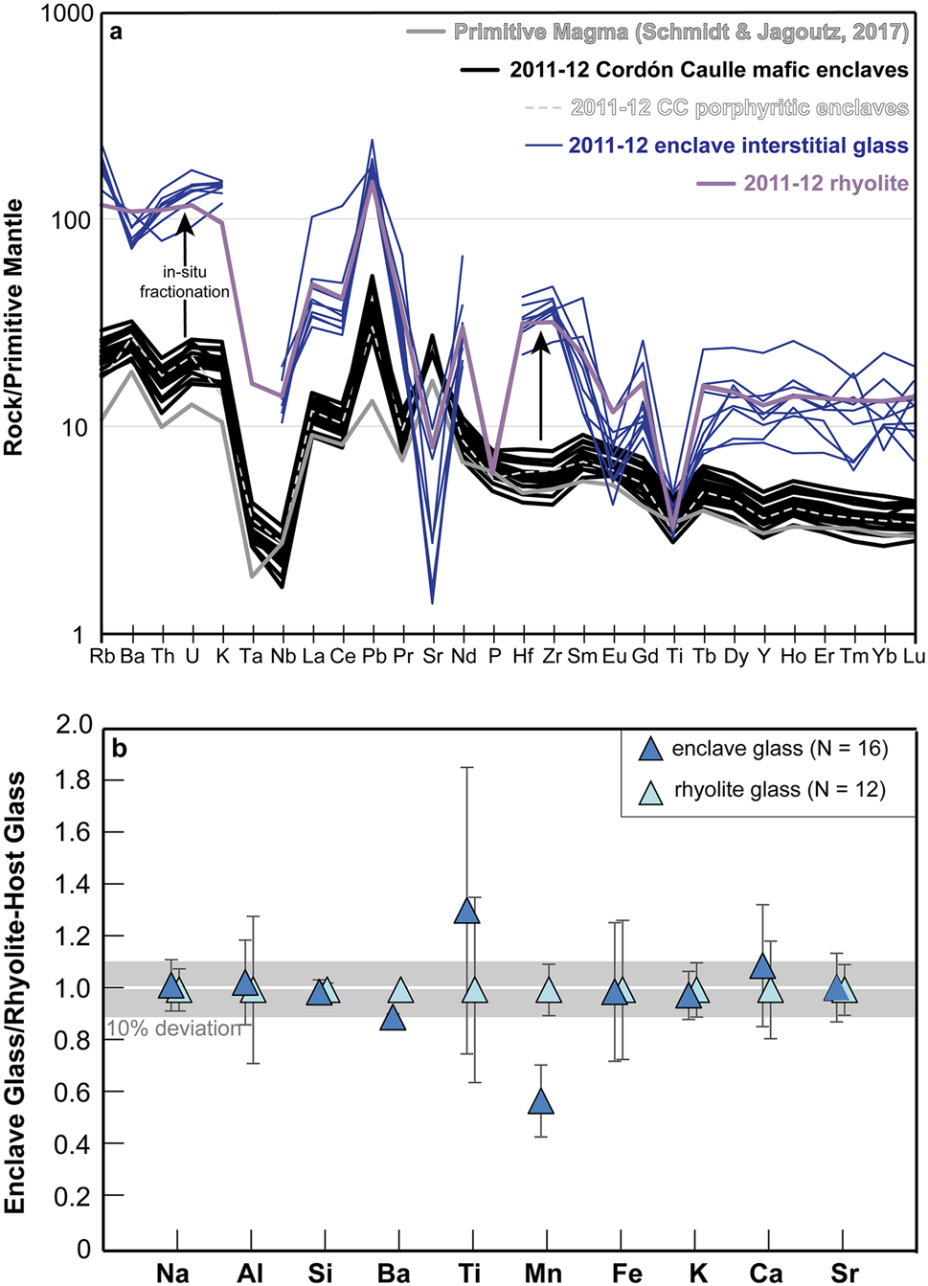
(**a**) Incompatible trace element spider diagram (XRF + ICP-MS data) normalized to primitive mantle^63^. Grey line: representative primitive magma^32^. Black line: 2011–12 CC coarsely-crystalline mafic enclaves. Dashed light grey line: 2011–12 CC porphyritic enclaves. Thin dark purple line: 2011–12 mafic enclave interstitial glass data (LA-ICP-MS). Light Purple: 2011–12 host rhyolite (XRF + ICP-MS). Arrows indicate symmetrical elevation of rhyolite data from mafic enclaves suggestive of in-situ fractionation. (**b**) 2011–12 CC mafic enclave interstitial glass normalized to host rhyolite glass and display overlapping compositions. Symbols are the same as Fig. 4.

The 2011–12 mafic enclaves from Cordón Caulle represent the primitive end-member for the entire eruptive history at PCC and are among the most primitive magmas for the entire SVZ (Fig. 4)^15^. With their range from 50 to 53 wt% SiO_2_, 5.29–9.3 wt% MgO, Mg#_~53–66_ (calculated on a molar basis with (Mg/(Mg + Fe) × 100), 22–113 ppm Ni, and 94–248 ppm Cr (Fig. 4), the most primitive enclaves have major element compositions comparable to other primitive and near-primitive magmas globally^32^. Whole-rock trace element data displays typical subduction zone signatures for the mafic enclaves (Fig. 5a). Additionally, the enclaves lack a Eu anomaly suggesting minimal plagioclase fractionation or accumulation (Fig. 5a). Subtle subgroupings of the enclaves can be identified based on major and trace element data but are not the focus of this study. To first order, Fe-enriched and Fe-depleted trends correlate with TiO_2_, Al_2_O_3_, and CaO trends (Fig. 4, ESM 6). TiO_2_ and Al_2_O_3_ display two distinct trends of enrichment and depletion, while CaO exhibits two clusters at ~ 12 wt% and ~ 10 wt% CaO (ESM 6).

The 2011–12 mafic enclaves are substantially more primitive in comparison to other well studied volcanic systems globally that have mafic enclaves with a range of enclave textures and eruptive environments. The 2011–12 mafic enclaves stand alone with uniquely elevated MgO, Mg#, Ni, and Cr contents (Fig. 4). Most other mafic enclaves are andesitic to dacitic. The closest analog to the 2011–12 enclaves are the most mafic enclaves from Medicine Lake, CA^33,34^. Both magmatic systems also share highly silicic host magmas (> 70 wt% SiO_2_) as well as an extensional tectonic environment. However, while Medicine Lake enclaves display a full range of enclave compositions from felsic to mafic, the 2011–12 mafic enclaves are exclusively mafic.

### Mineral compositions

#### Plagioclase

Core analyses for plagioclase phenocrysts in the coarsely crystalline enclaves are dominantly anorthitic (An_80–92.3_), and only in rare cases do core compositions drop to ~ An_76_ (ESM 1, 7). Plagioclase mantle compositions cluster around An_55–65_ but fully range from An_41.4–72.6_. Detailed rim analyses were not performed, but the thin (< 10 µm) rims have an approximate composition of An_40–45_. Those rim compositions correlate with the plagioclase compositions in the rhyolite^17^. The porphyritic enclave population has plagioclase phenocrysts with a more restricted calcic compositional range (An_85.8–92.5_) and rims range from An_39–41_. Microlite cores, excluding the porphyritic population, cluster from An_80–88_ with a full range from An_71.5–88.5_. The more sodic rims cluster from An_55-65_ but have a full range from An_36.4–68.9_. The microlite cores from the porphyritic population are more restricted with a compositional range from An_89.4–91.62_ and rims from An_60.9–70.6_.

#### Olivine and clinopyroxene

Olivine core compositions from coarsely-crystalline enclaves range from Fo_~70–85.7_ with rims at Fo_~58–63.9_ (ESM 1, 7). Olivine from porphyritic enclaves is on average more magnesian (Fo_79.2–86.4_) with extensive flat cores. We did not resolve core-rim compositional changes in the thin olivine rims. Clinopyroxene phenocrysts from the coarsely-crystalline and the porphyritic enclaves cluster at Mg#_75–85_ (full range Mg#_55.6–83.6_) and Mg#_64.5–69_, respectively (ESM 1, 7).

## Discussion

### Mafic injection vs crystal mush

Mafic enclaves are commonly interpreted as products of mafic magma injection into a more silicic magma, but that is not the only explanation for their origin. Alternatively, mafic enclaves can represent fragments of cumulates or crystal mushes. In the case for magma injection origin, porphyritic textures and microstructural evidence supports formation in a dynamic liquid-rich environment^1,25,28^. The thermal contrasts between the injected magma and the silicic host magma result in large cooling rates and produce quench textures, especially along the enclave margins, associated with diffusion-limited crystal growth^28^. Alternatively, mafic enclaves that form from cumulates or crystal mushes will be dominated by interlocking textures of larger grains growing under interface-controlled conditions during slow continuous cooling in a rigid crystal network^28,35^.

#### Coarsely-crystalline enclaves

Multiple lines of evidence support a crystal mush origin for the coarsely-crystalline enclaves. They are highly crystalline (55–70%), display interlocking cumulate grain textures, show dominantly simple mineral zonation patterns with a range in An and Fo content, contain angular void filling vesicles, and are chemically distinct compared to the global compilation of other mafic enclaves (Fig. 4; ESM 6). The large phenocryst sizes (300–600 µm), planar faces, and interlocking textures suggest prolonged and stable crystal growth that is possible in a mush environment^28,36,37^. Equant crystal shapes with planar faces, especially prevalent in plagioclase, are indicative of slow cooling rates (as opposed to elongate grains that result from rapid undercooling)^29^. However, a lack of grain orientations or textural lineation amongst any of the enclaves implies accumulation or compaction from crystal settling is not prevalent^28^. Liquid-rich magma chambers, especially low viscosity mafic magmas, are ideal environments for crystal settling and accumulation that produce undeformed, euhedral grains that are aligned in shape-preferred orientations^28^. The coarsely-crystalline enclaves, however, display randomly oriented and intergrown phenocrysts and microlites. Such textures are characteristic of crystal-rich environments like that of a crystal-rich mush^28^. Melt pockets are irregularly shaped around the edges of phenocrysts faces which suggests their shape is controlled by the highly crystalline network, and their lack of parallel sides further argues for the lack of influence from compaction. Additionally, angular-shaped void filling vesicles suggest that gas exsolution occurred in the presence of a rigid crystal mush framework and formed where space was available (Figs. 2, 3; ESM 3–5)^38,39^. Simple zonation patterns in phenocrysts, especially plagioclase, provide evidence for storage in a thermally and compositionally buffered system that was not disturbed by recharge events of differing composition. Injection-sourced enclaves typically exhibit disequilibrium textures and chemical zoning representative of mixing and homogenization which we do not observe. Mafic recharge commonly produces a large thermal contrast between the injection and host magma resulting in oscillatory zoning and quench textures^25^. Minor evidence of reactive processes exists in the form of heterogeneous strands of groundmass within the rhyolite surrounding the enclave-rhyolite interface indicative of minimal interaction (ESM 5). The angularity of the coarsely-crystalline enclaves, pressure shadows in the host rhyolite, and lack of significant chilled margins preclude melt- or liquid-dominated incorporation mechanisms such as magma injection. Instead, these characteristics suggest that the enclaves were already largely crystallized with a more or less rigid crystalline matrix when they were entrained within the rhyolite.

**Figure 6:**
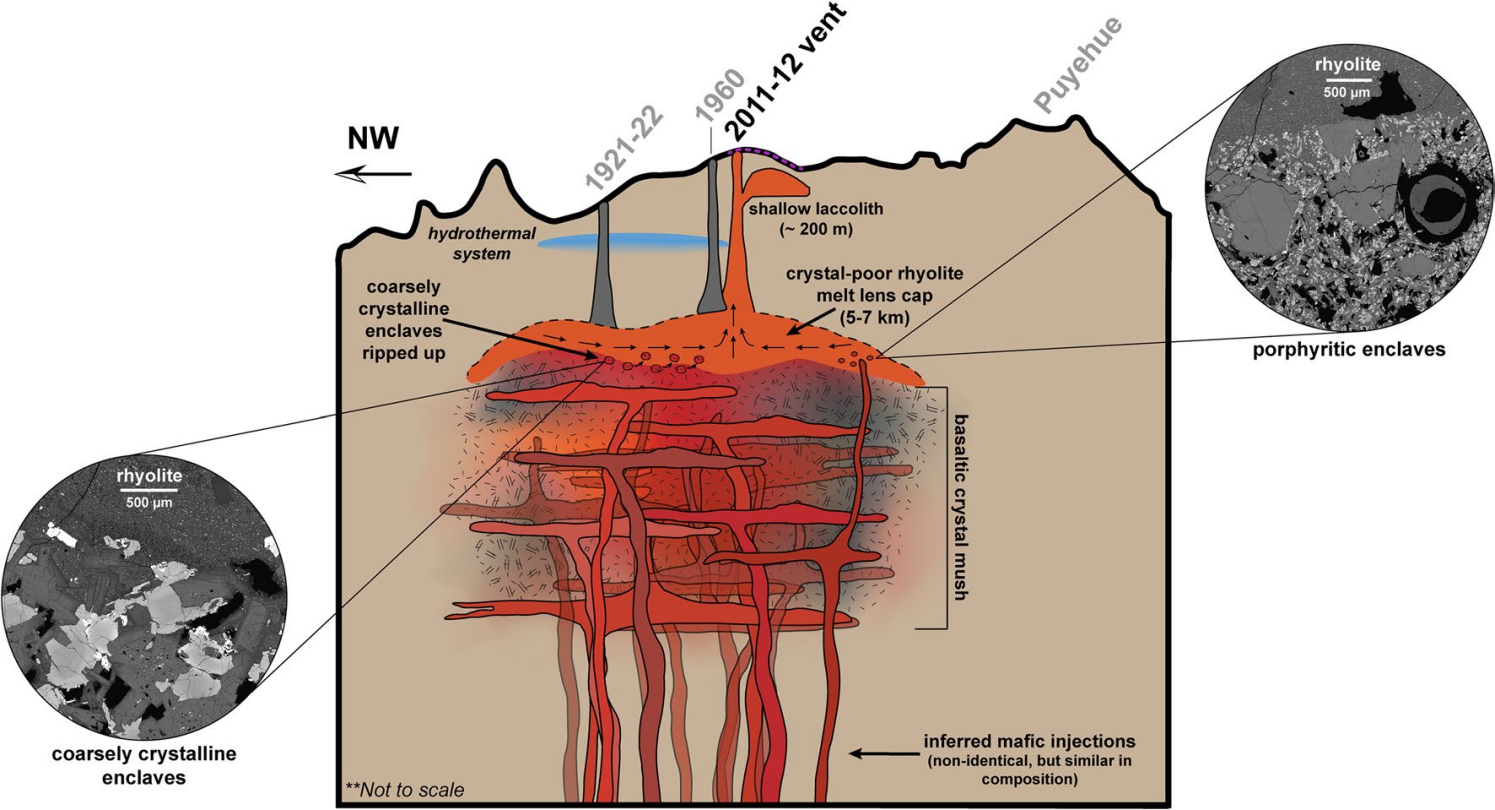
Schematic diagram for Cordón Caulle plumbing system (not to vertical scale). Inferred mafic injections^22^ fuel overlying crystal mush. Shades of orange-red in mafic injections and crystal mush represent compositional heterogeneity observed in chemistry. Stippled texture in crystal mush represents different levels of crystallinity. Black portions of crystal mush represent low-melt content or inactive heterogeneities within the mush. Crystal mush is overlain by crystal-poor rhyolite (orange). Coarsely-crystalline fragments are entrained into rhyolite prior to or upon eruption preserving remnants of the mush in the form of mafic enclaves. Some injections bypass the mush chemically unobstructed and interact directly with rhyolite to form porphyritic enclave population. Arrows in rhyolite melt cap represent June 4–7, 2011 magma flux^22^. Dashed purple line: surficial uplift from laccolith emplacement. Circular outsets are BSE images of enclave-host boundary for both enclave populations. Schematic does not infer magmatic architecture underlying Puyehue volcano.

Assuming the coarsely-crystalline enclaves represent a crystal mush, we propose their incorporation occurred during eruptive withdrawal of the overlying crystal-poor magma that entrained fragments of the underlying crystal mush (Fig. 6). If that was the case, the rigid nature of the crystalline matrix and the spatial proximity of the mush to the overlying rhyolite ensured that textures and compositions were only weakly overprinted during entrainment and transport. Thermal differences are likely small if the crystal-poor rhyolite directly overlies the mush. Given the rigid, crystal-rich mush, entrainment is likely inhibited, resulting in a relatively small abundance of entrained mush, and hence minimal abundance of enclaves within the erupting magma. The 1921–22 and 1960 eruptions produced nearly identical rhyolite lavas to the 2011–12 rhyolite, and thus all eruptions are suggested to be sourced from the same reservoir^17,18,22^. However, mafic enclaves are only identified in the 2011–12 eruption. We envision these three eruptions are progressively tapping the rhyolitic melt lens cap without fully depleting the reservoir, and therefore, the most recent eruption (2011–12) is able to reach the interface between the rhyolite melt lens cap and the basaltic crystal mush (Fig. 6). We place the melt lens above the crystal-rich mush without significant overlying crystal-rich regions due to the absence of mafic enclaves in the prior two eruptions (1921–22, 1960). Eruption of the crystal-poor rhyolite from a melt lens within a crystal-rich mush should result in prevalent enclave incorporation within all three eruptions as erupting magmas would have to transit the mush.

To further corroborate the presence of a crystal mush, the coarsely-crystalline enclaves must show a genetic relationship to the rhyolite that overlies the mush. We attribute the formation of the rhyolite to fractional crystallization of the crystal-rich mush. Major elements such as MgO, FeO, Al_2_O_3_, K_2_O, TiO_2_, P_2_O_5_ support this notion with non-linear, fractionation trends between the enclaves, interstitial glass, and rhyolite compositions as opposed to linear mixing trends (Fig. 4; ESM 6). Incompatible trace elements within the rhyolite are uniformly elevated relative to the enclaves, consistent with in-situ fractionation, thus suggesting a genetic relationship between the enclaves and host rhyolite (Fig. 5a). Furthermore, incompatible trace elements of the enclave interstitial glass, collected via LA-ICP-MS, have similar compositions and compositional trends as the whole-rock rhyolite (Fig. 5a). Lastly, glass data from the interstitial melt of the enclaves and glass from the rhyolite have nearly identical compositions (Fig. 5b), aside from Ti and Mn. Variations in Ti are large for both enclave and rhyolite lithologies and are likely affected by small variations in oxide abundances. Reasons for the deviations in Mn are not known. The overlapping glass data between the enclaves and rhyolite suggest a genetic relationship between the two lithologies, where interstitial melt from the crystal-rich mush is extracted to form the overlying melt lens cap. Thus, we interpret the rhyolitic interstitial melt of the mafic enclaves to represent the interstitial melt of a basaltic crystal mush that feeds a rhyolitic melt lens that was tapped in the 2011–12 eruption. We propose that at Cordón Caulle, rhyolite formation is short-circuited via highly efficient fractionation directly from a basaltic mush (Figs. 5, 6). This contrasts the typical, protracted differentiation paths that require smaller and progressive jumps in composition where rhyolite is produced from an intermediate crystal-rich mush^5–7^. This will be discussed in more detail in the “Compositional Gap” discussion section.

#### Porphyritic enclaves

The low crystallinity, porphyritic texture, disequilibrium textures (swallowtails) on plagioclase, abundant microlites, and a narrow range in mineral chemistry of the 2011–12 porphyritic enclaves is consistent with an injection-sourced enclave model that is envisioned for numerous other enclave-bearing magmatic systems^1,4,25,33,34,40–47^. Similar to the porphyritic enclaves at Cordón Caulle, those systems have enclaves with crystallinities of ~ 10–30%, porphyritic textures, evidence of rapid cooling in the form of quenched or glassy margins, and a range in crystal sizes with abundant microlites near enclave margins. A major difference for Cordón Caulle enclaves, is their rare abundance (< 1 vol%) compared to a much greater enclave abundance in other systems (> 1–10 vol%)^1,3,25^. Thus, while these enclaves may be part of the eruption-triggering mechanism, e.g. tipping a system already in a near-critical state, they are unlikely to be the singular driver, especially as the dominant coarsely-crystalline enclaves are crystal mush related and not attributed to injection.

Instead, the porphyritic enclaves provide more information about magma assembly and the sub-surface architecture. The presence of isolated phenocryst clusters within the groundmass are indicative of varying crystal nucleation and growth rates. The swallowtail disequilibrium textures on plagioclase phenocrysts that are bordered by groundmass suggest a change in crystal growth environment from interface-controlled slow growth to diffusion-limited rapid growth, likely due to increased undercooling (Fig. 3i, ESM 2e,f)^29,48^. Injection of a magma containing phenocryst clusters into a cooler host rhyolite led to rapid quenching and a shift in crystal growth dynamics, resulting in the observed swallowtail disequilibrium textures. Additionally, the large spherical vesicles (~ 700–1000 µm) that are unbound by phenocryst phases (Fig. 3g) suggest there was not a pre-existing crystal network and bubble growth was able to occur in a liquid-dominated system^38,39^. Groundmass crystallization appears to have been subsequent to bubble formation.

Although we link the porphyritic enclaves texturally to magma injection, the enclaves lack chemical evidence for mixing or hybridization during interaction with the crystal-rich magma mush or rhyolite lens. Such interactions are common in enclave-bearing systems^45,49^ and predicted when recharge and mush compositions are similar^50^. Mixing leads to diverse crystal populations that are distinct in composition, size, and internal zonation^51,52^. In contrast, olivine and plagioclase compositions from the porphyritic enclaves are tightly bound (~ Fo_84_ and ~ An_85.8–92.5_ (ESM 1, 7), when compared to phenocrysts in the coarsely-crystalline enclaves. This suggests that in rare occasions mafic injections bypass the mush chemically unobstructed and become entrained in the rhyolite upon eruption. Their low abundance suggests that such pathways are either rare or that the individual volume of mafic injections is small compared to the shallow crystal mush reservoir. We speculate that the crystal-rich mush beneath the rhyolite lens is spatially and thermally heterogeneous creating areas of higher and lower melt content, where inactive portions of the mush have a lower melt content and higher crystallinities that lead to a more rigid behavior amenable to fracturing. This would allow for some of the mafic injections to travel through the mush without mixing or hybridization, producing the tightly bound primitive compositions in olivine within the porphyritic enclaves. Ultimately these injections reach the rhyolite chemically unobstructed, thus entraining primitive porphyritic enclaves in the eruption alongside the coarsely-crystalline enclaves (Fig. 6).

### The importance of melt extraction and mush heterogeneity

We defer to a crystal mush origin for the coarsely crystalline enclaves as opposed to cumulate origin due to their textures and chemistry. While crystal mush systems can foster accumulation signatures due to crystal settling and melt extraction^53,54^, this would lead to preferred crystal orientations, foliation around large grains, frequent glomerocrysts, and minimal microlites typical for cumulates^28^. Moreover, those processes produce bulk chemistry changes in response to the preferential accumulation or removal of mineral phases such as olivine and plagioclase. This causes, for example, elevated MgO and Ni concentrations and Eu anomalies, respectively. The mafic enclaves in the 2011–12 eruption have neither typical cumulate textures nor do they show chemical evidence for accumulation of olivine or plagioclase (Fig. 5a). Instead, their composition lacks an Eu anomaly and they are more akin to primitive magmas (5–9 wt% MgO)^32^. Thus, we interpret the Cordón Caulle enclaves to be representative of a crystal mush that has not undergone large degrees of crystal settling nor preserves signatures of significant melt extraction. To produce large volumes of silicic magma by fractionation from a crystal mush without significantly altering the mush composition, requires a sufficiently large mush. In case of a several kilometer thick mush, the upper parts of the mush could be in a compositional steady state with silicic magma removal upward and replenishment of mafic magma from below retaining its basaltic nature and overprinting signatures of significant melt removal. Such a conceptual model would also imply that the mush is spatially contiguous with the overlying rhyolite. The crystal mush could become progressively more cumulate-like with depth, but the coarsely-crystalline enclaves only represent the top layers of the crystal mush (Fig. 6).

While the coarsely-crystalline enclaves are considered a cohesive population, they do encompass a range of compositions in major and trace elements (MgO, Mg#, Ni, Cr, Al_2_O_3_, CaO), which is also reflected in their range in mineral chemistry. The dispersed range in olivine Fo content (Fo_~70–85.7_) and plagioclase An content (An_~80–92_) in the coarsely-crystalline enclaves indicate a composite nature of the enclaves that likely reflects subordinate compositional heterogeneity within the mush. We interpret that lower Fo olivines represent the long-lived mush, whereas the higher Fo olivines provide evidence for mafic additions that sustain the mush thermally and by mass. In other words, the mush may be episodically recharged by mafic injections that compositionally resemble the porphyritic enclaves. Mush heterogeneity is sustained through compositionally variable mafic recharge and evolve locally to small varying degrees depending on the injection history and thermal state (Fig. 6). We envision episodic mafic injections into the base of the mush that permeate the system or primitive melts that get injected into the mush at various depth levels. These injections keep the crystal mush basaltic, thermally buffered to prevent complete solidification, and sustain the overlying rhyolitic system at shallow levels^17,18,20^.

### Compositional gaps

**Figure 7:**
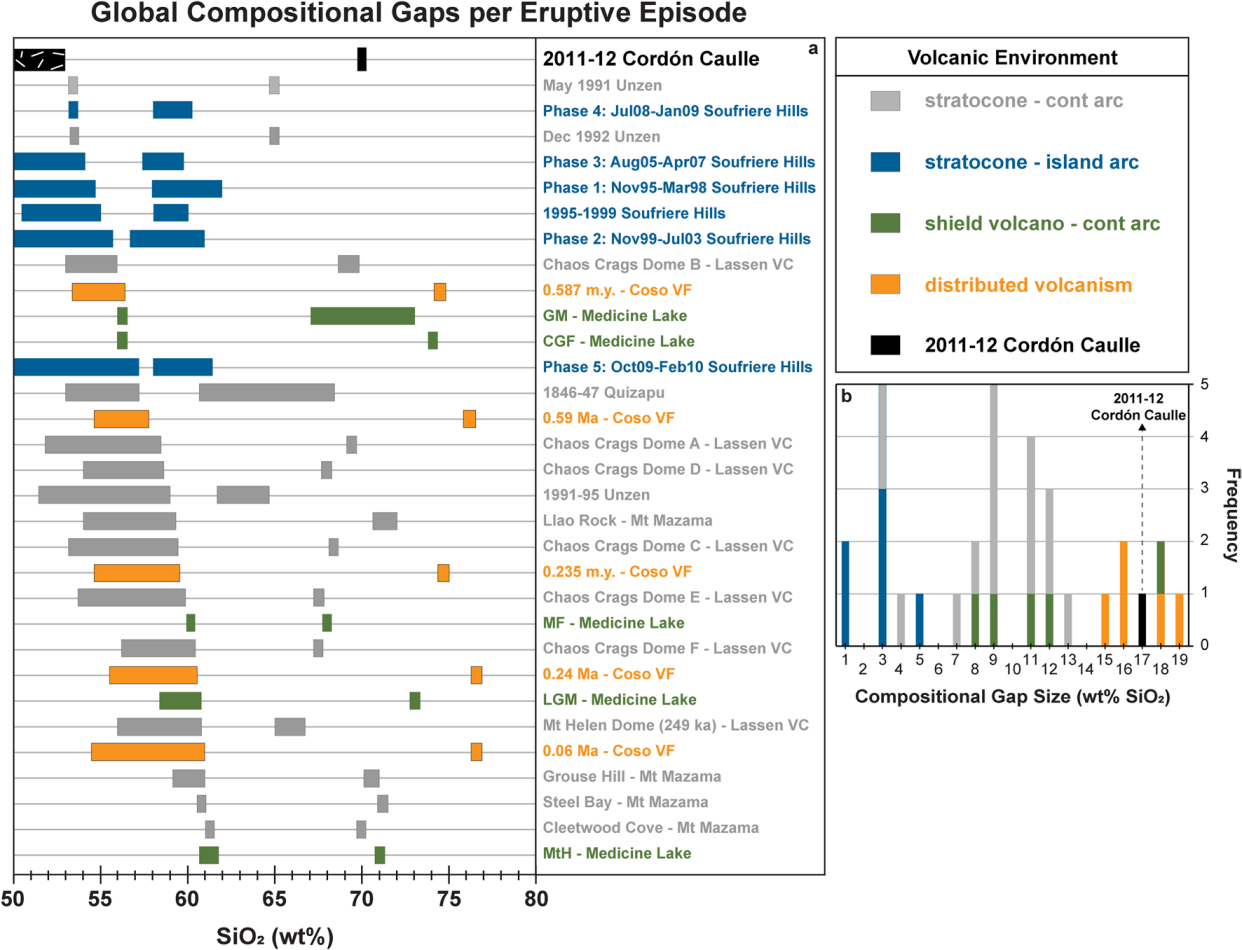
(**a**) Compositional gap data for 2011–12 Cordón Caulle eruption compared to global eruptions with compositional gaps between enclaves and host lavas. (**b**) Frequency diagram of compositional gap magnitude. 2011–12 CC eruption produced 17 wt% SiO_2_ compositional gap. For typical on-arc systems, compositional gaps range from < 1 to 14 wt% SiO_2_ making CC an end-member. Figure is color coordinated to volcanic environment. Black: 2011–12 Cordón Caulle eruption (continental on-arc system). Grey: stratocones/continental on-arc systems. Blue: stratocones/island arc systems. Green: shield volcano/continental arc system. Orange: distributed volcanism. References for compiled eruptions located in ESM 1.

The 2011–12 Cordón Caulle eruption produced an extreme compositional gap, and thus may inform us about the full spectrum of differentiation processes common to compositional gaps^12,13^. A meaningful comparison of compositional gaps needs to be addressed at the level of individual eruptions, therefore, we compiled global data sets of mafic enclaves hosted in silicic lavas from individual eruptions at a range of tectonic and volcanic settings (Fig. 7). The full range of compositional gaps in our compilation is < 1 wt% SiO_2_ up to 18 wt% SiO_2_, but rarely exceeding 16 wt% SiO_2_. The 2011–12 eruption produced one of the largest compositional gaps at 17 wt% SiO_2_. For stratocones and other arc systems the global range is restricted to < 1–14 wt% SiO_2_ making Cordón Caulle’s gap an extreme end-member for on-arc magmatic systems. Moreover, the 2011–12 enclaves are the most mafic of all mafic end-members associated with compositional gaps, thus raising the question why mush dynamics (i.e. crystal-melt separation) are so effective in magma differentiation (evidenced through fractional crystallization from basalt to rhyolite) at Cordón Caulle compared to other systems with smaller compositional gaps and less mafic end-members^5–8,28,55–57^.

Cordón Caulle demonstrates that crystal mush arc systems can produce rhyolite directly from basalt as the chemistry between rhyolite and the interstitial enclave glass are identical and fractionation trends characterize the overall major and trace element compositions of the 2011–12 eruption (Figs. 4, 5; ESM 6). Multiphase-fluid dynamic modeling suggests that crystal-melt separation becomes highly efficient at ~ 50–70% crystallinity in response to rheologic lock up which is observed at Cordón Caulle^13^. However, compaction, a commonly invoked mechanism^5^, alone cannot produce the volume of melt via crystal-melt segregation observed at Cordón Caulle within the 40-year recurrence interval^17,18,22^. Based on the compaction equations (Eqs. 5–9) from Bachmann and Bergantz^5^, we used a range of reasonable values for the height of mush layer (20–440 m), porosity (0.4), solid phase density (2844 kg/m^3^), liquid phase density (2350 kg/m^3^), dynamic melt viscosity (3.47 × 10^4^ Pa s), grain size (0.005 m radius), permeability (1.904 × 10^−8^m^2^), bulk and shear viscosity of the matrix (1 × 10^−14^ Pa s), and compaction length (1.13 × 10^–13^ m), and a crystal mush always requires a minimum of ~ 265 years to produce enough melt for the 2011–12 eruption^5^, not even addressing the need to produce rhyolite for three similarly sized eruptions in less than 100 years. Alternatively, the abundant presence of vesicles in the enclaves may point toward gas sparging as a mechanism to fractionate melt from the crystal framework^8,58^. Lastly, future poro-elastic modelling of ground deformation from the 2011–12 eruption could point toward magma reorganization implying pressure changes influenced melt separation from the crystal-rich mush^24^. Cordón Caulle provides an opportunity to further test whether an individual mechanism dominates melt extraction or whether a combination of mechanisms is needed to produce such large compositional gaps.

The compositional gap at Cordón Caulle most closely resembles the gap at Medicine Lake, CA, which has been explained by late-stage fractionation (57–61% solids removed) at shallow pressures (1–2 kbar) via a nearly flat cotectic during crystallization^33^. In their study, the solidus–liquidus line can flatten in a temperature-composition projection that allows for large amounts of crystallization to occur over a small temperature window resulting in a drastic compositional change in the residual liquid. Ultimately, Grove and Donnelly-Nolan^33^ demonstrated that for calc-alkaline systems, increased crystallization may occur over a minimal temperature interval that allows for significant compositional gap formation. However, the Medicine Lake compositions involve amphibole that is absent in the 2011–12 enclaves, the latter being consistent with tholeiitic magmas typical for Puyehue-Cordón Caulle. Nonetheless, the interlocking textures of plagioclase, olivine, and clinopyroxene suggest coeval crystallization followed by late-stage orthopyroxene reaction rims in the coarsely-crystalline enclaves and may point towards similar processes effective at Medicine Lake explaining the formation of a such a large compositional gap. The primitive nature of microphenocrysts in the coarsely-crystalline enclaves suggests late-stage melt evolution and is consistent with late-stage fractionation to produce the rhyolite melt during shallow storage (3–7 km depth)^17,18,20^. The 2011–12 Cordón Caulle eruption may represent an alternative to the common protracted model of silicic magma production along a transcrustal evolutionary path with continual differentiation as magmas migrate upward in the crust^7^. Instead, Cordón Caulle provides evidence for differentiation within the shallow crust that short-circuits multi-stage processing.

## Conclusion

While the rhyolitic eruptions from Cordón Caulle have been studied in great detail, our study reports previously undescribed basaltic enclaves that provide a more complete picture of the magmatic system. We interpret the coarsely-crystalline mafic enclaves hosted in rhyolite lava as evidence for a compositionally zoned magmatic system comprised of a crystal-rich basaltic, but internally-heterogeneous, magma mush overlain by a crystal-poor rhyolite melt lens cap. The porphyritic enclaves are interpreted to represent open-system behavior that generates rare mafic magma injection directly into the rhyolitic melt lens cap. Enclave chemistry supports the notion that the crystal-poor rhyolite is genetically related to the mafic enclaves through highly efficient fractionation via melt extraction from the crystal-rich mush. The efficient extraction of interstitial melt from the mush has produced one of the largest compositional gaps globally at 17 wt% SiO_2_, with near-primitive to primitive compositions directly producing rhyolite at shallow depths without any involvement of intermediate compositions. The implications of the mafic enclaves and their connection to the erupted rhyolite challenge the multi-stage, transcrustal evolutionary path for silicic magma generation that typically produces a range of compositions. Cordón Caulle’s spatially dispersed eruptive vents and distributed ground deformation throughout the graben are consistent with our interpretation of a laterally extensive crystal mush storage reservoir, and our work on the mafic enclaves provides direct evidence for the presence of a crystal mush at an active system. Additionally, our identification and analysis of the enclaves determined the direct source of the silicic magma generation for the 2011–12 eruption. In short, we provide geochemical and textural evidence that the erupted rhyolite was sourced from the interstitial melt of the basaltic crystal mush and was extracted to form an eruptible melt lens cap. Findings from these enclaves will also inform past and future interpretations of geophysical and geodetic observations at Cordón Caulle.

## Methods

### Sampling and enclave characterization

A total of 33 mafic enclaves were collected along most of the periphery of the 2011–12 flow field as well as in the interior of the flow near the crater vent (Fig. 1). While rare (~ < 1 vol%), the enclaves are almost ubiquitously dispersed, and in a few areas we find them in clusters of up to ~ 10 enclaves within a few meters of each other. A systematic assessment of their size, abundance, and distribution, and whether smaller enclaves (< 1 cm) are more abundant, cannot be assessed due to the overall scarcity of enclaves. Brief reconnaissance work of the 1921–22 and 1960 lavas has not revealed enclaves in these earlier flows confirming previous work that did not report enclaves in these lavas.

Crystallinity, phase percentages, and vesicularity estimates are based on image analysis using JMicroVision. Randomized point-counting (n = 500) was conducted with uncertainties of ~ 5% based on counting statistics. Crystallinity estimates are based on phenocrysts which we define as crystals > 100 µm. Microlites in the groundmass are defined as < 100 µm. Additionally, this study refers to melt and groundmass where melt refers to crystal-free (including microlite-free) liquid, while groundmass includes microlites. The terms melt and glass are used interchangeably.

### Whole-rock and mineral chemistry

Bulk rock major and trace elements were determined for 19 mafic enclaves via X-ray fluorescence and inductively-coupled plasma mass spectrometry at the GeoAnalytical Lab at Washington State University following the analytical procedure of Johnson et al.^59^ (ESM 1A). A duplicate bead was made to assess the analytical reproducibility. Major element data reports R^2^ = 1.0 and trace element data reports R^2^ = 0.99.

Major and trace element concentrations in plagioclase, olivine, clinopyroxene, oxides, and glass were determined on eight samples via a JEOL JXA-8200 electron microprobe equipped with five wavelength dispersive spectrometers and a silicon-drift energy dispersive spectrometer at Washington University in St. Louis. A mean atomic number background method was used^60^. Complete run conditions, elements analyzed, and reference materials are all reported in ESM 1B-G. 2-point core-rim analyses were performed on plagioclase, olivine, and clinopyroxene and single spot analysis were used for oxides and glass pockets. Plagioclase and glass measurements were performed with a defocused beam of 20 µm and Na was measured first to avoid Na loss.

Trace element concentrations of enclave interstitial melt were collected via laser ablation inductively coupled mass spectrometry (LA-ICP-MS) at the University of Nevada, Reno following the procedure of Woodhead et al.^61^. Data was reduced using Iolite software^62^. LA-ICP-MS was conducted on the coarsely-crystalline enclaves. A total of 38 singular points were collected at 33 µm diameter: 11 points in the enclave core interstitial melt, 10 points in the enclave-host transition interstitial melt, 3 points in the enclave-host transition groundmass, and 14 points in rhyolite groundmass. Reference material, elements analyzed, uncertainty, and analytical conditions are reported in ESM 1H,I.

## Supplementary Information

Supplementary Information 1.

Supplementary Information 2.
