# Supplementary material for: Insights for crystal mush storage utilizing mafic enclaves from the 2011–12 Cordón Caulle eruption
Source: Sci Rep. 2022 Jun 13;12:9734. doi: 10.1038/s41598-022-13305-y (PMC9192606; doi:10.1038/s41598-022-13305-y)
Supplement: Supplementary file 2 — Supplementary Information 2. [file 41598_2022_13305_MOESM2_ESM.pdf]

Supplementary Materials

ESM 1. Excel file with all analytical conditions and raw data collected for this study as well as list of references for global enclave compilation and compositional gap per eruption.

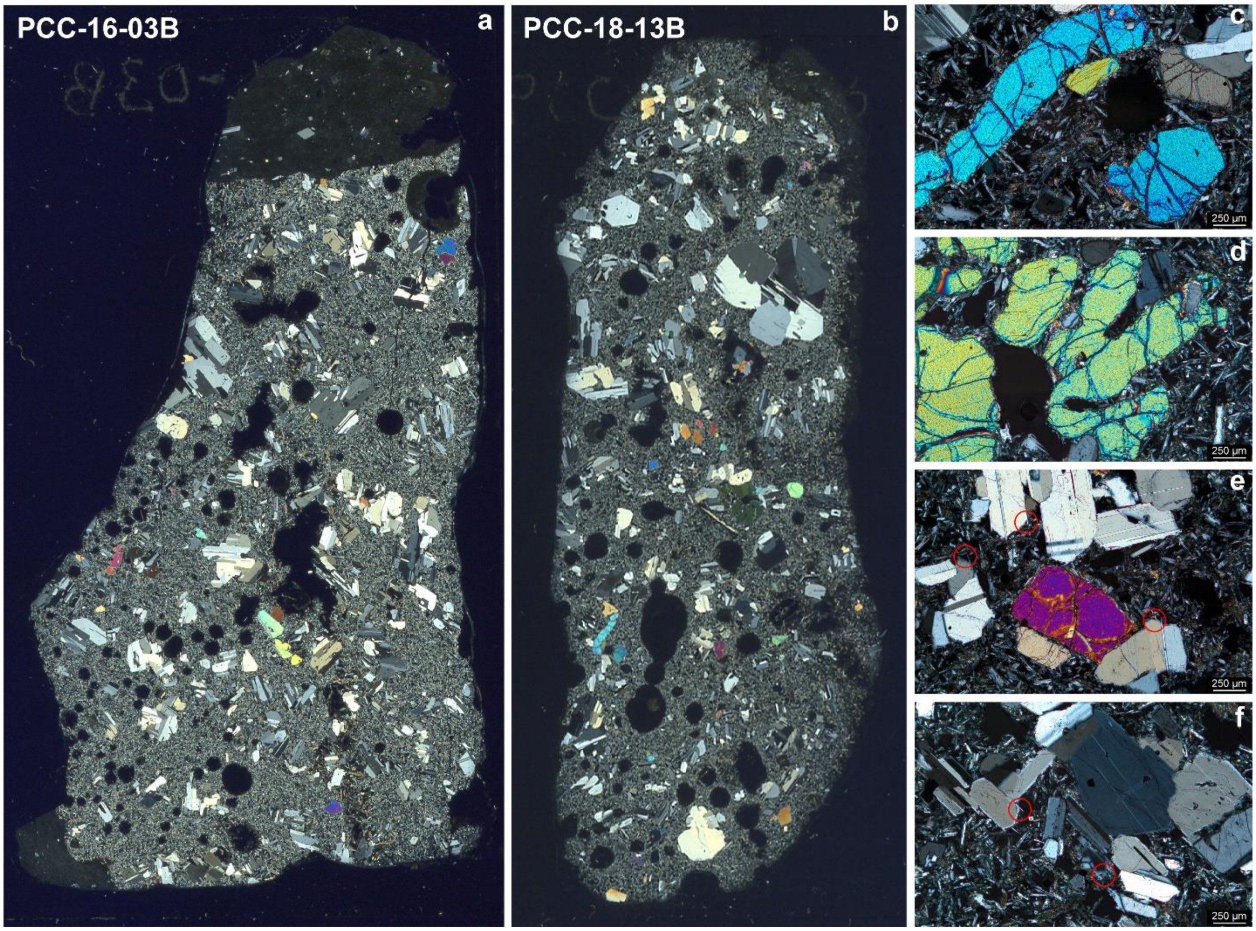

ESM 2. Textural images of the porphyritic enclave population. (a-b) Thin section scans of porphyritic enclaves highlighting crystallinity, glomerocrysts, mineralogy, and vesicle shape. (c-d) Micrographs of skeletal olivine texture. (e-f) Micrographs of plagioclase glomerocrysts. Red circles highlight swallowtail disequilibrium texture.

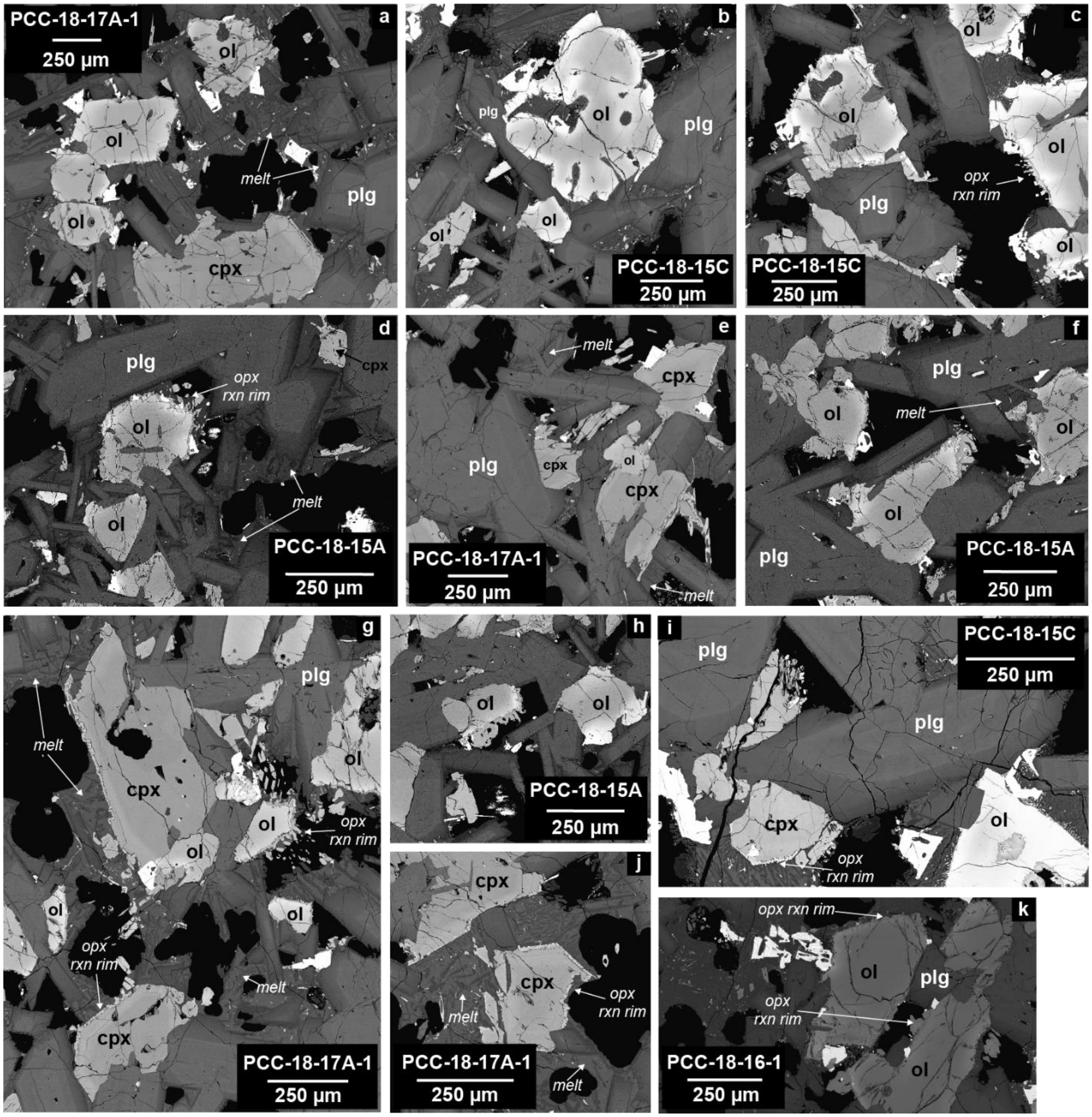

ESM 3. BSE images of coarsely crystalline enclaves. Panels highlight intergrown textures, plagioclase zonation, and plagioclase resorption/sieve textures. Plagioclase zonation “core-mantle-rim” notated in panel (b, g, i).

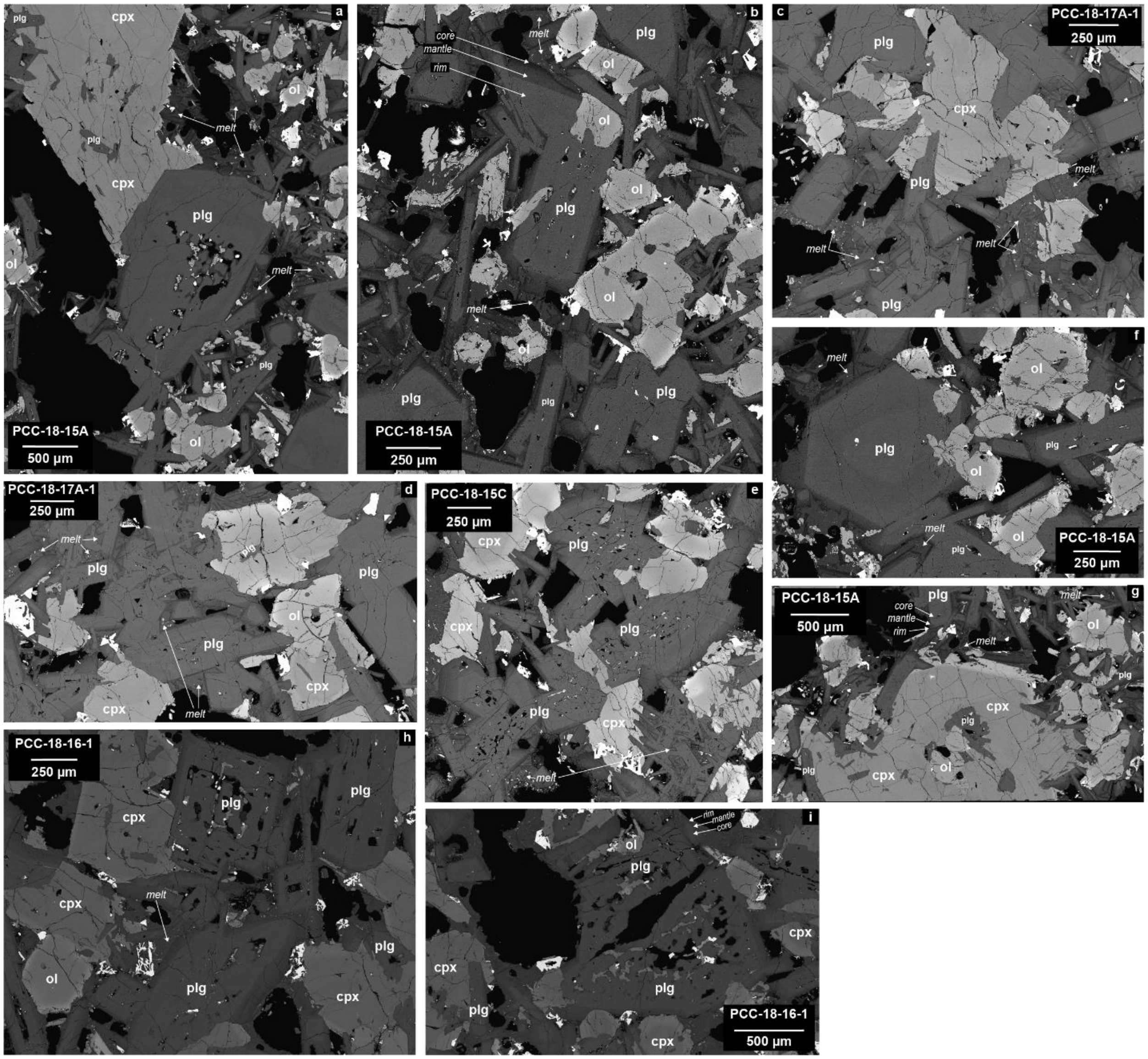

ESM 4. BSE images of coarsely crystalline enclaves. Panels highlight orthopyroxene reaction rims on olivine and clinopyroxene. “Opx rxn rim” notated on panels (c, d, g, j, k).

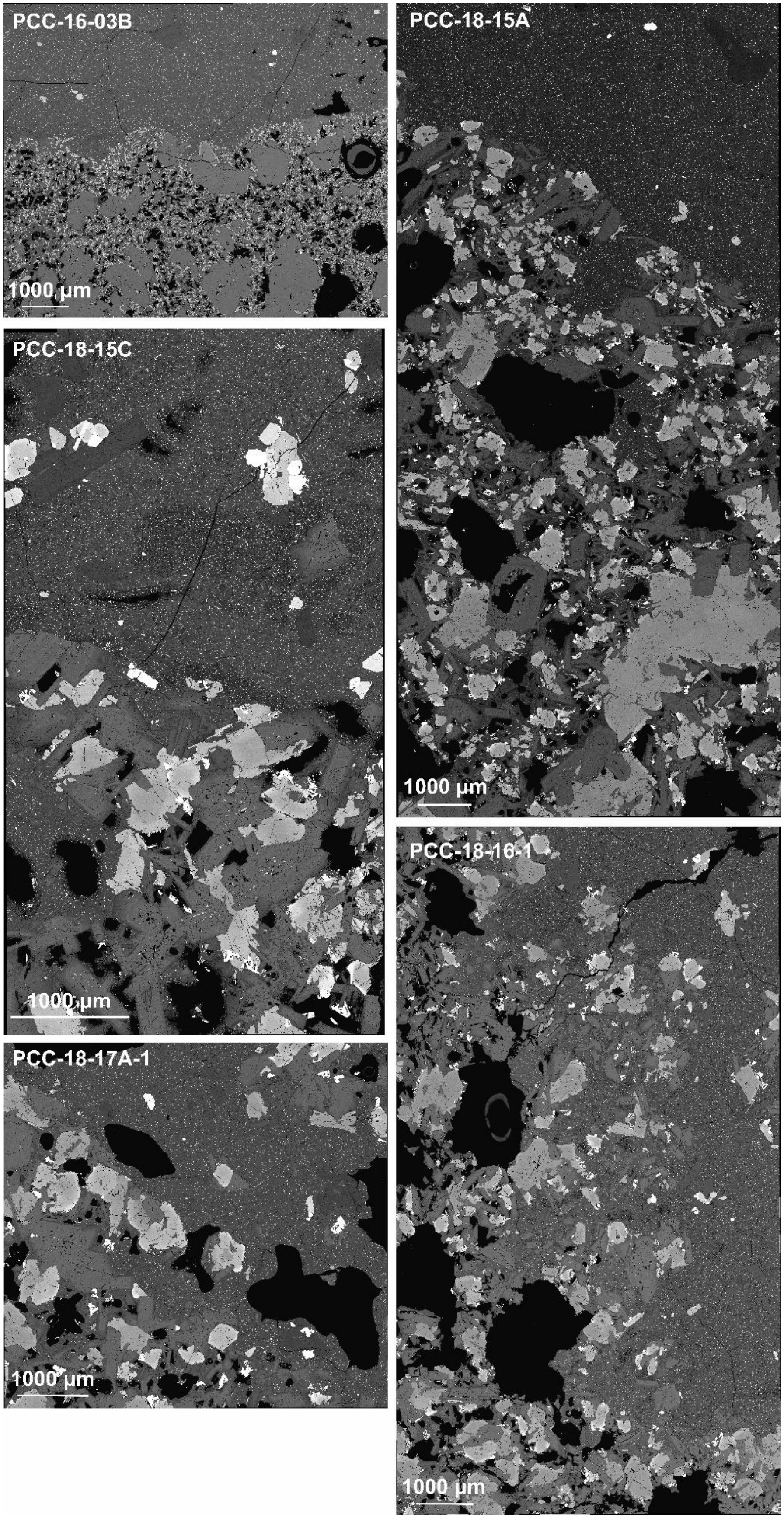

ESM 5. BSE maps of enclave-host boundary. Sample PCC-16-03B is the porphyritic population and the rest are of the coarsely crystalline population. PCC-18-15C displays heterogeneous strands in groundmass at boundary (dark grey vs light grey).

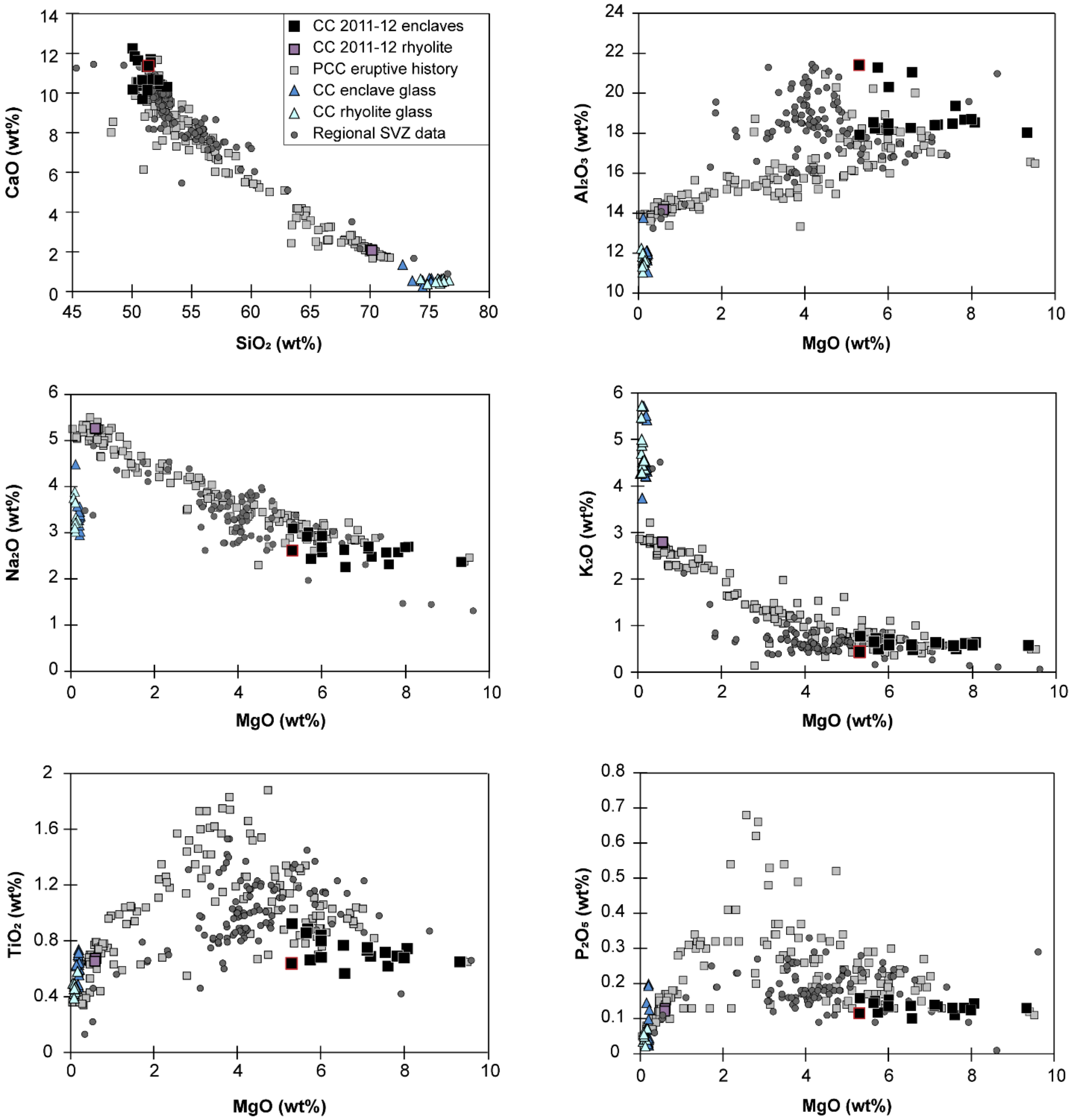

ESM 6. Geochemical Harker diagrams. Symbols are the same as Fig. 4.

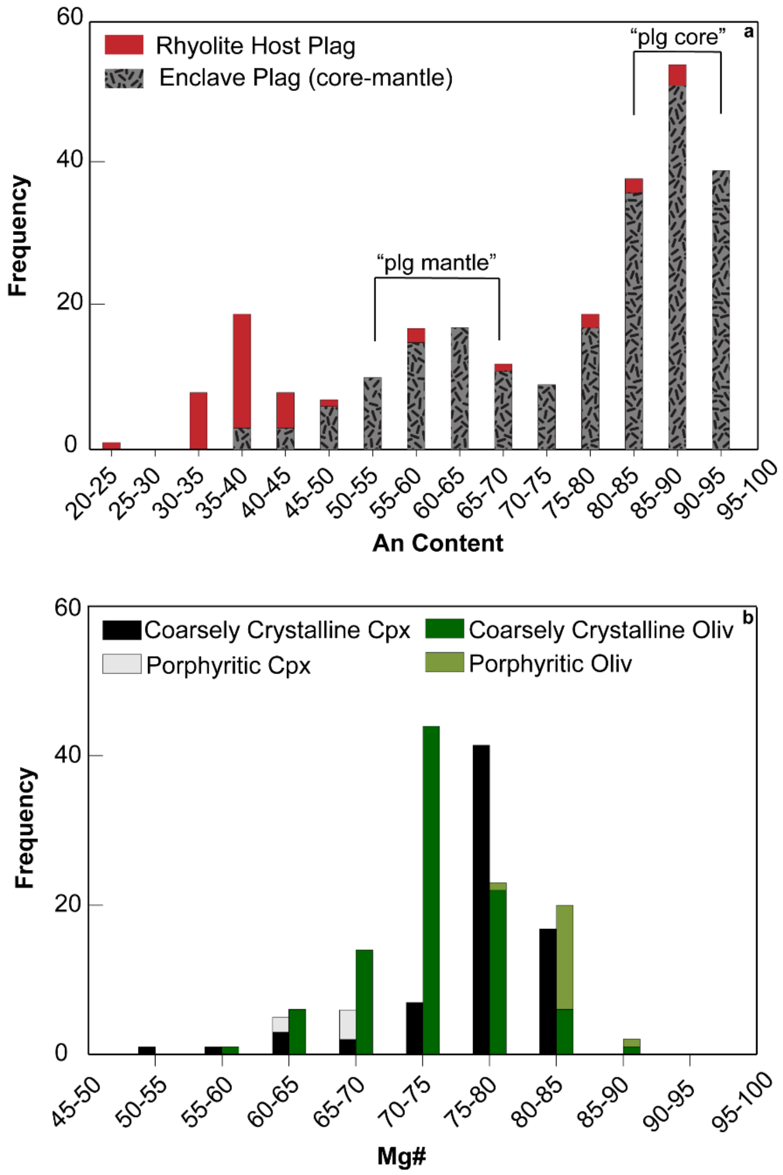

ESM 7. Mineral geochemistry for plagioclase, olivine, and clinopyroxene. (a) An content for plagioclase in both enclave populations. Frequency bins are 5 mol%. Plagioclase core-mantle zonation labeled for coarsely crystalline enclaves. (b) Mg# frequency bins for olivine and clinopyroxene. Dk green: Olivine in coarsely crystalline population. Lt green: Olivine in porphyritic population. Black: Cpx coarsely crystalline population. Lt grey: Cpx porphyritic population. Porphyritic olivine displays tightly bound range at higher Mg# compared to coarsely crystalline olivine.
